# Relationship between the perception of disrespectful treatment and abuse during childbirth and the risk of postpartum post-traumatic stress disorder: a PPQ-based study

**DOI:** 10.3389/fgwh.2025.1568446

**Published:** 2025-05-22

**Authors:** Inmaculada Ortiz-Esquinas, Ana Rubio-Álvarez, Ana Ballesta-Castillejos, Julián Rodríguez-Almagro, Juan Miguel Martínez-Galiano, Antonio Hernández-Martínez

**Affiliations:** ^1^ Department of Obstetrics and Gynecology, Reina Sofia University Hospital, Córdoba, Córdoba, Spain; ^2^ Department of Obstetrics and Gynecology, Torrejón University Hospital, Madrid, Spain; ^3^ Department of Nursing, Faculty of Nursing of Ciudad Real, University of Castilla-La Mancha, Ciudad Real, Spain; ^4^ Department of Nursing of University of Jaen, Jaén, Spain; ^5^ Consortium for Biomedical Research in Epidemiology and Public Health (CIBERESP), Madrid, Spain

**Keywords:** abuse, obstetric violence, post-traumatic stress disorder, postpartum, women's mental health

## Abstract

**Problem:**

Childbirth is often portrayed as a positive and empowering experience, yet for many women, it can result in negative emotional outcomes, which may contribute to the development of postpartum Post-Traumatic Stress Disorder (PTSD). Understanding the association between perceived abuse during childbirth and PTSD is crucial for improving maternal care.

**Background:**

Research shows that disrespectful and abusive treatment during childbirth is linked to psychological distress and PTSD. However, the correlation between perceived abuse and PTSD in postpartum women remains underexplored.

**Aim:**

To determine the association between the woman's perception of abuse during childbirth and the risk of developing PTSD postpartum, as well as to analyze related risk factors.

**Methods:**

A cross-sectional observational study was conducted with 2,912 women in Spain who gave birth in the last 18 months. The Childbirth Abuse and Respect Evaluation- Maternal Questionnaire (CARE-MQ) assessed perceived abuse, while the Perinatal PTSD Questionnaire (PPQ) measured PTSD risk. Logistic regression was used to adjust for confounders.

**Findings:**

Higher CARE-MQ scores were positively correlated with PTSD risk, especially in the “inadequate treatment by professionals” dimension (*r* = 0.56). Extreme perception of abuse (≥95th percentile) increased the PTSD risk (aOR = 34.72). Additional risk factors included extremely premature birth, unrespected birth plans, complications, type of birth and emergency cesarean sections.

**Discussion:**

Perceived mistreatment and inadequate professional care strongly correlate with PTSD risk.

**Conclusion:**

Addressing these factors—along with other identified risks—may help reduce PTSD prevalence and improve maternal care experiences.

## Introduction

Childbirth is a transcendental event in women's lives. How this is carried out, as well as the attention and care provided during it, can affect the recovery and experience of the postpartum, with important repercussions on the maternal emotional area, on the mother-child relationship, and in the care that the mother provides to the newborn (1–3). For this reason, in 2018, the World Health Organization (WHO) proposed a model of childbirth care focused on women and their babies, seeking to integrate perinatal mental health into maternal and childcare. This model prioritizes the birth experience as a fundamental element to guarantee quality care, which means creating a safe environment from a clinical and psychological point of view that ensures the highest degree of maternal well-being and satisfaction in relation to her birth experience (4).

These practices include excessive or unnecessary medicalization, non-consensual or inappropriate interventions—such as performing episiotomies without consent, conducting painful procedures without anesthesia, or forcing women to give birth in a specific position—as well as physical or verbal abuse, limitation of autonomy, and lack of emotional support or adequate information. This form of violence can also be psychological, taking the shape of infantilizing, paternalistic, authoritarian, humiliating, or degrading treatment, including verbal insults, depersonalization, or mocking behavior.

Furthermore, in recent years, various international institutions (5, 6) and social groups (7, 8) have revealed a substantial growth in practices and behaviors on the part of health professionals, which, both by action and omission, are disrespectful in terms of physical and emotional aspects. These practices include excessive or unnecessary medicalization, non-consensual or inappropriate interventions—such as performing episiotomies without consent or conducting painful procedures without anesthesia—as well as physical abuse, limitation of autonomy, and lack of emotional support or adequate information. This form of violence can also be psychological, taking the shape of infantilizing, paternalistic, authoritarian and humiliating treatment (9–11). This phenomenon is known as obstetric violence (OV), and it is a form of institutional violence against women, as well as an important public health problem (12). Due to the lack of a clear consensus on its definition, as it is a multidimensional and complex phenomenon, a variety of approaches and approximations has been proposed, making data collection and subsequent analysis difficult.

Even so, various studies examining the prevalence and manifestations of obstetric violence have reported that between 21% and 81% of the women surveyed have experienced at least one form of obstetric violence (11). In Spain, we find disparity in prevalence according to studies, but it is estimated between 38.3% and 67.4% (13, 14).

This form of violence may be perceived by women as a traumatic experience during perinatal care, especially during childbirth, with the consequent impact on their postnatal emotional health and increased risk of various conditions, including post-traumatic stress disorder (PTSD) (10, 15). It is estimated that PTSD related to childbirth affects approximately 4.7%–11% of postpartum women in high-income countries. For instance, a recent systematic review and meta-analysis by Heyne et al. (16), which included studies conducted primarily in European and other high-income contexts, found an overall estimated prevalence rate of 4.7% in mothers, with the time postpartum assessed ranging from 1 to 14 months. Additionally, a validation study conducted in Spain by Hernández-Martínez et al. (17) using the Perinatal Post-Traumatic Stress Disorder Questionnaire (PPQ) reported a PTSD risk prevalence of approximately 11% among Spanish postpartum women. In high-risk groups, such as women with a history of preterm birth, stillbirth or preeclampsia, the prevalence increases to 15.7% (18). It is essential to keep in mind that maternal perception determines what constitutes a vital risk for her and her baby. Therefore, even a birth that seems normal or without complications from an obstetric point of view can be experienced as traumatic (15, 19). Nonetheless, there are known prenatal vulnerability factors (history of previous trauma, history of anxiety or depression), as well as intrapartum risk factors (unwanted medical interventions, lack of emotional support, birth experience) that are related to a higher risk of developing postpartum PTSD (15, 20).

PTSD, which can appear up to a year after giving birth, is characterized by the appearance of various symptoms that include the appearance of intrusive memories (flashbacks, nightmares), avoidance attitudes (avoiding talking or avoiding certain places), changes in mood, as well as increased irritability, difficulty concentrating and tokophobia (15, 20). All of this causes significant distress for women and can lead to poor adaptation to motherhood and serious imbalances in the emotional development of the newborn and even in the establishment and maintenance of breastfeeding (15, 21, 22).

Given the negative consequences it has on women and their families, it is important to understand and address the relationship between PTSD and abuse during childbirth. Knowing the associated risk factors could help us plan lines of care and care guides during pregnancy and childbirth that are favorable for maternal postpartum mental health.

## Materials and methods

### Study design and subject selection

A cross-sectional observational study was conducted with postpartum women whose birth took place from June 2022 to December 2023 throughout Spain. This study was approved by the clinical research ethics committees of the Hospitales Mancha-Centro, Hospital Universitario Reina Sofía de Córdoba, and Hospital Universitario de Ciudad Real. All participants received written information about the study and signed the informed consent prior to their participation. The informed consent form was signed electronically and collected through the same online survey, ensuring the participants' voluntariness and consent before their inclusion in the study.

The exclusion criteria were women under 18 years of age with an inability to read and speak Spanish (language barrier).

The questionnaire was distributed to 3,043 women, of which 4.3% (131) of the women did not agree to participate in our study.

The maximum modeling criterion was used to estimate the sample size, which requires including 10 subjects for each independent variable (23). Taking into account that the risk prevalence of PTSD risk could be up to 11% (17), 200 women at risk of PTSD and a total of 2,000 women are required to include a minimum of 20 independent variables in the multivariate analysis.

### Information sources

To collect the required information, an online questionnaire was distributed to associations related to pregnancy, birth, and postpartum, as well as to breastfeeding support groups throughout the Spanish territory. The questionnaire included sociodemographic variables, obstetric history, variables of the most recent birth, obstetric practices carried out, and neonatal results. This questionnaire had previously been piloted in a sample made up of women of different cultural levels, ages, and economic levels and from different geographical areas.

After applying the inclusion and exclusion criteria, the participating women were informed and accepted informed consent for participation in the research, providing a contact telephone number or email address.

Various tools were included in this questionnaire:

• Childbirth Abuse and Respect Evaluation- Maternal Questionnaire (CARE-MQ), version 2. This tool is made up of Likert-type questions about different practices and/or situations that can be related to abuse and lack of respect during childbirth. The possible answers are: “It did not occur during my birth” (0 points), “It occurred, but it did not affect me” (1 point), “It occurred, and it affected me a little” (2 points) and “It occurred, and it affected me a lot” (3 points). The total score ranged from 0 points to 60 points. The scores can be categorized according to the distribution of percentiles (≤50th percentile—Low Risk, 51–75th percentile—Medium Risk, 75–90th percentile—High Risk, 90–95th percentile—Very High Risk, ≧95th percentile—Extreme Risk). The tool has shown adequate internal consistency and excellent temporal stability in test-retest. The Childbirth Abuse and Respect Evaluation—Maternal Questionnaire (CARE-MQ) was validated by Hernandez et al. in 2024, in English and Spanish versions, to assess women's perceptions of abuse and/or disrespect they may have experienced during childbirth in a Spanish postpartum population (24).
• Perinatal Post-Traumatic Stress Disorder Scale (PTSD) Questionnaire (PPQ). The risk of PTSD was assessed using the Perinatal Post-Traumatic Stress Disorder Questionnaire (PPQ).

This questionnaire has been validated and used in a population similar to that of the study. This tool consists of 14 questions with Likert-type responses with scores ranging from 0 to 56 points (17). We considered a high-risk score for post-traumatic stress disorder as a score equal to or greater than the 90th percentile of its distribution. “Emotional Abuse” was measured with items 7, 10, 11, 12, 13, and 14 of the questionnaire, while “Inadequate Professionalism” was measured with items 1, 2, 3, 4, 5, 8, 9, 15, and 18. “Physical Abuse” was measured with questions 16, 17, and 19, and the fourth dimension, “Lost Contact,” was measured with items 6 and 20.

### Statistical analysis

First, a descriptive analysis was performed; for qualitative variables, absolute and relative frequencies were used, and for quantitative variables, the mean and standard deviation (SD).

Next, the bivariate relationship between the CARE-MQ scale as a whole and its dimensions with the PPQ scores as a whole was studied using the Pearson correlation coefficient. The next step was to determine the relationship of each CARE-MQ item with the PPQ score using analysis of variance (ANOVA) to determine which aspects present a higher average PTSD risk score.

Finally, the relationship between the perception of abuse and disrespect during childbirth was analyzed using the CARE-MQ scale (grouped in percentiles), and the risk of PTSD using the PPQ scale (a score equal to or greater than the 90th percentile of its distribution). The multivariate analysis also included all potential confounding factors. Crude (OR) and adjusted Odds Ratios (aOR) were estimated with their respective 95% confidence intervals (95%CI) using binary logistic regression (Backward Stepwise Regression).

## Results

### Sample characteristics

A total of 2,912 women participated with a mean age of 33.69 years (SD = 4.03 years), and 77.9% (2,267) were primiparous. Almost all (94.8%, 2,761) were full-term pregnancies (≥37 weeks), and almost half (45.9%, 1,338) of the cases labor was induced. 59.1% (1,720) of women were administered oxytocin during labor, and 82.1% (2,390) used regional analgesia. Women at risk for post-traumatic stress disorder (PTSD) (>90th percentile score) encompassed 10.1% (293) of cases, with a mean PPQ score of 11.44 (SD = 12.34). 5.2% (152) presented extreme levels of perception of abuse during childbirth (≧95th percentile) and the mean CARE-MQ score was 7.72 points (SD = 10.38). The remaining characteristics of the sample are shown in Table 1.

**Table 1:** Sample characteristics.

Variable	N (%)	Mean (DE)
	*N* = 2,912	
Score CARE- MQ		7.72 (10.38)
CARE-MQ (Grouped by percentiles)		
Percentile ≤50 (3 points) low	1,339 (46.0)	
Percentile 51–75 (3–11 points) medium	807 (27.7)	
Percentile 76–90 (11–22 points) high	453 (15.6)	
Percentile 91–94 (22–31 points) very high	161 (5.5)	
Percentile >95 (≧31 points) extreme	152 (5.2)	
PPQ score		11.44 (12.34)
Risk of PTSD (PPQ > 90 percentile)		
No	2,619 (89.9)	
Yes	293 (10.1)	
Age		33.69 (4.03)
Family income		
<1,000 euros	44 (1.5)	
1,000–1,999 euros	512 (17.6)	
2,000–2,999 euros	1,038 (35.6)	
3,000–3,999 euros	806 (27.7)	
≥4,000 euros	512 (17.6)	
Perception of amount of partner support		
None	49 (1.7)	
Little	71 (2.4)	
Some	181 (6.2)	
Sufficient	705 (24.2)	
A lot	1,906 (65.5)	
Planned pregnancy		
No	204 (7.0)	
Yes	2,708 (93.0)	
Live fetus		
No	15 (0.5)	
Yes	2,897 (99.5)	
Gestational age		
Term	2,761 (94.8)	
Moderate-late premature (32−36 + 6 weeks)	133 (4.6)	
Very premature (28−31 + 6 weeks)	6 (0.2)	
Extremely premature (<28 weeks)	12 (0.4)	
Type of gestation		
Single	2,861 (98.3)	
Multiple	49 (1.7)	
Missing	2	
Previous cesarean		
No	2,168 (74.5)	
One	692 (23.8)	
Two or more	52 (1.8)	
Number of pregnancies		
One	1,840 (63.2)	
Two	781 (26.8)	
Three or more	291 (10.0)	
Number of vaginal births		
None	622 (21.4)	
One	1,808 (62.1)	
Two or more	482 (16.6)	
Number of miscarriages		
None	2,105 (72.3)	
One	596 (20.5)	
Two or more	211 (7.2)	
Hypertension		
No	2,652 (91.1)	
Yes	260 (8.9)	
Diabetes		
No	2,680 (92.0)	
Yes	232 (8.0)	
Spontaneous preterm birth		
No	2,744 (94.2)	
Yes	168 (5.8)	
Fertility treatment		
No	2,523 (86.6)	
Yes	389 (13.4)	
Parity		
Primiparous	2,267 (77.9)	
Multiparous	645 (22.1)	
Antenatal classes		
No	458 (15.7)	
Yes, but less than 5 classes	561 (19.3)	
Yes, at least 5 classes	1,893 (65.0)	
Induction of labor		
No	1,574 (54.1)	
Yes	1,338 (45.9)	
Birth plan		
No	1,208 (41.5)	
Yes, but it wasn't respected	475 (16.3)	
Yes, and it was mostly respected	1,229 (42.2)	
Health problem in last pregnancy		
No	2,219 (76.2)	
Yes	693 (23.8)	
Use of oxytocin to stimulate labor		
No	1,192 (40.9)	
Yes	1,720 (59.1)	
Regional		
No	522 (17.9)	
Yes	2,390 (82.1)	
Nitrous oxide		
No	2,823 (96.9)	
Yes	89 (3.1)	
General		
No	2,776 (95.3)	
Yes	136 (4.7)	
Type of delivery		
Normal	1,655 (56.8)	
Instrumental	598 (20.5)	
Planned cesarean section	127 (4.4)	
Emergency cesarean section	532 (18.3)	
Episiotomy		
No	2,271 (78.0)	
Yes	641 (22.0)	
Severe tear		
No	2,775 (95.3)	
Yes	137 (4.7)	
Skin-to-skin following birth		
No	534 (18.3)	
Yes, but less than 50 min	333 (11.4)	
Yes, between 50 and 120 min	336 (11.5)	
Yes, at least 120 min	1,709 (58.7)	
Breastfeeding within first hour		
No	792 (27.2)	
Yes	2,120 (72.8)	
Neonatal admission		
No	2,524 (86.7)	
Yes, in the Neonatal ICU	166 (5.7)	
Yes, but not in Neonatal ICU	222 (7.6)	
ICU admission		
No	2,864 (98.4)	
Yes	48 (1.6)	
Readmission following discharge		
No	2,837 (97.4)	
Yes	75 (2.6)	
Surgical intervention after birth		
No	2,751 (94.5)	
Yes	161 (5.5)	
PTS (PPQ > 90 percentile)		
No	2,619 (89.9)	
Yes	293 (10.1)	

### Correlation between the dimensions of the CARE-MQ questionnaire and the PPQ questionnaire scores

**Figure 1:**
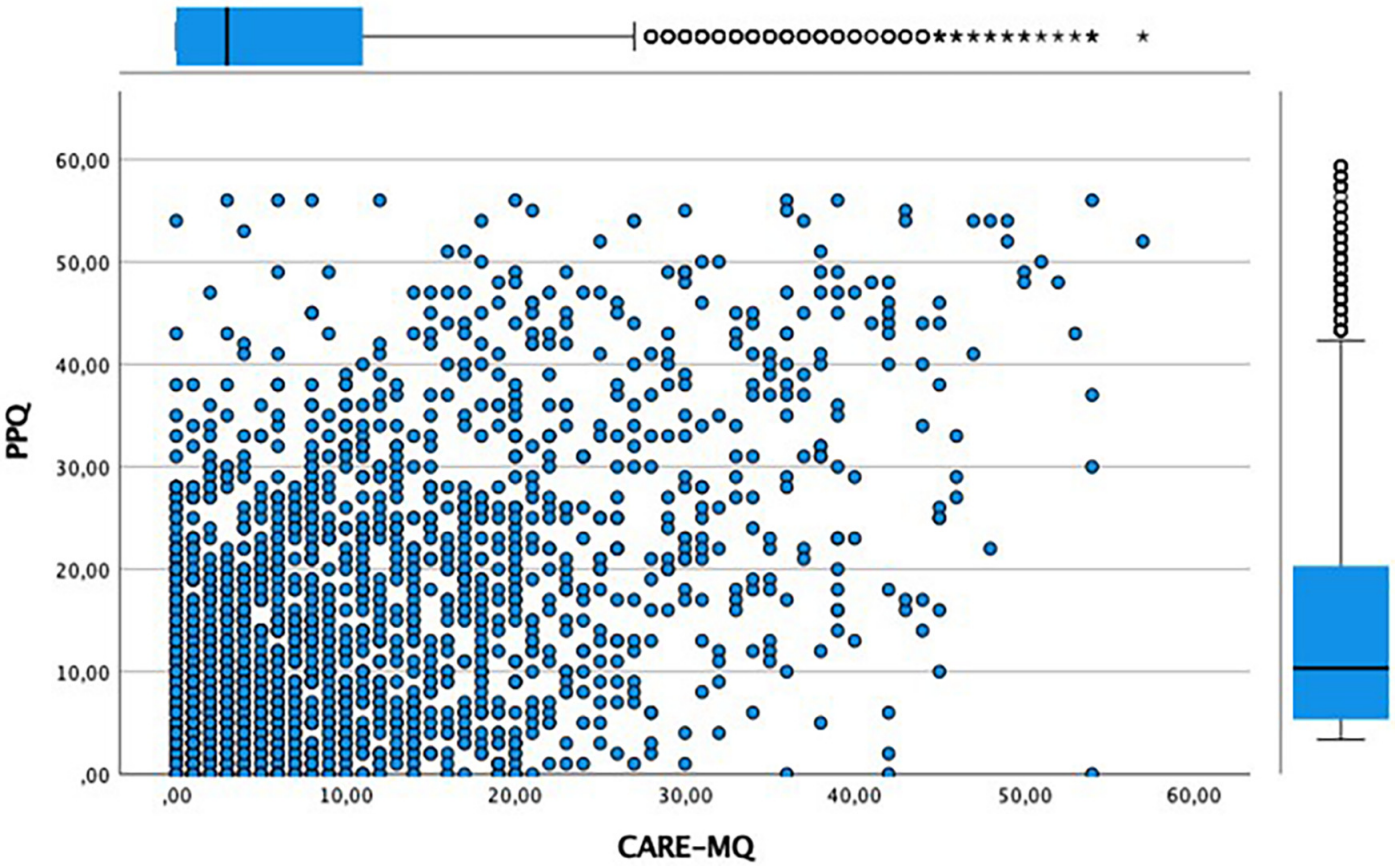
Relationship between the scores on the CARE-MQ and the PPQ scales.

The correlation between the dimensions of variation of the CARE-MQ questionnaire and the scores of the PPQ questionnaire was analyzed. “Emotional abuse” with *r* = 0.47 (95%CI: 0.44–0.50), “inadequate treatment by professionals” with *r* = 0.56 (95%CI: 0.54–0.59), “physical abuse” *r* = 0.36 (95%CI: 0.33–0.40) and “separation” *r* = 0.39 (95%CI: 0.36–0.42), correlate positively (*p* < 0.001) with PTSD, with inadequate treatment by professionals being the element that most correlate. This relationship can be observed graphically in Figure 1.

### Relationship between CARE-MQ scores and PPQ scores

Next, to analyze the differences in scores between the CARE-MQ scale and the PPQ scores, an analysis of variance (ANOVA) was performed, observing a statistically linear trend in all items *p* < 0.001. Thus, the higher the scores on the CARE-MQ scale, the higher the scores on the PPQ scale, thus the greater the risk of post-traumatic stress disorder. The item that presented the highest average score was Item 19 related to physical violence. As seen in Table 2, those women who were very affected had an average PPQ score of 33.9 points (SD = 17.31).

**Table 2:** Relationship between the dimensions of the CARE-MQ questionnaire and the PPQ questionnaire scores.

	(0 points)	(1 point)	(2 points)	(3 points)	*P* value
	Mean (SD)	Mean (SD)	Mean (SD)	Mean (SD)	
Items on information received from the professionals (Items 1–3)	Information received	Information not given, but it did not affect me AT ALL	Information not given, and it affected me A LITTLE	Information not given, and it affected A LOT	
1. The professionals that assisted at my birth introduced themselves by name and profession	9.12 (10.70)	13.85 (12.61)	19.19 (13.81)	25.82 (16.77)	**<0** **.** **001**
	It occurred	It occurred, but it did not affect me AT ALL	It occurred, and affected me A LITTLE	It occurred, and affected me A LOT	
2. They explained to me the techniques and/or procedures that were going to be performed on me (for example, placing an IV, rupturing the amniotic sac, administering medication, etc.) and the reason why, the alternatives, as well as the risks and benefits of them in an understandable way, and/or I was able to ask the questions that arose and choose between the proposed alternatives	8.58 (9.87)	11.95 (10.85)	16.59 (13.12)	27.07 (15.28)	**<0**.**001**
	It occurred	It occurred, but it did not affect me AT ALL	It occurred, and affected me A LITTLE	It occurred, and affected me A LOT	
3. They explained clearly how my labor was progressing, or my health status, or that of my infant, in a way that I could understand and/or I was able to ask any questions I had	8.39 (9.46)	10.48 (9.77)	15.88 (12.90)	27.21 (15.46)	**<0**.**001**
Items regarding privacy (Items 4–5)	It occurred	It occurred, but it did not affect me AT ALL	It occurred, and affected me A LITTLE	It occurred, and affected me A LOT	
4. The professionals who treated me protected my privacy (using screens, covering my private parts, etc.)	9.53 (10.81)	14.39 (12.73)	19.12 (14.14)	27.29 (16.32)	**<0**.**001**
	It occurred	It occurred, but it did NOT affect me AT ALL	It occurred, and affected me A LITTLE	It occurred, and affected me A LOT	
5. During vaginal examinations and/or techniques, there were more people present than necessary (other doctors, nurses, orderlies, cleaning staff, etc.) or students (nursing, medicine) were present without anyone having asked my permission.	9.43 (10.95)	12.08 (10.88)	16.56 (13.35)	26.20 (16.08)	**<0**.**001**
Items regarding professional support and care received (6–9)	They allowed me	They did not allow me, but it did not affect me AT ALL	They did not allow me, and it affected me A LITTLE	They did not allow me, and it affected me A LOT	
6. I was allowed to be accompanied by the person I chose during the entire birth process	10.11 (11.28)	13.01 (11.43)	14.33 (12.61)	23.60 (15.89)	**<0**.**001**
	Yes, I was assisted	I was not assisted, but it did not affect me AT ALL	I was not assisted, and affected me A LITTLE	I was not assisted, and affected me A LOT	
7. When I requested help (to move, wash myself, pain relief, etc.) I was NOT assisted.	9.88 (11.07)	15.35 (14.20)	18.62 (13.65)	25.17 (15.85)	**<0**.**001**
	They helped me and answered my questions	They did not help me, but it did not affect me AT ALL	They did not help me, and it affected me A LITTLE	They did not help me, and it affected me A LOT	
8. I was helped with care of my newborn, breastfeeding or artificial feeding, and they did NOT answer my questions	8.95 (10.29)	14.64 (13.76)	14.96 (13.50)	21.34 (15.32)	**<0**.**001**
	Yes, they respected it	They did not respect i, but it did not affect me AT ALL	They did not respect it, and it affected me A LITTLE	They did not respect it, and it affected meA LOT	
9. The professionals respected my birth plan when possible and when not possible they explained the reason to me and we agreed on an alternative	8.57 (9.73)	11.97 (11.14)	18.48 (13.88)	26.18 (15.15)	**<0**.**001**
Items regarding inadequate interpersonal relationship (Items 10–14)	It occurred	It occurred, but it did NOT affect me AT ALL	It occurred, and affected me A LITTLE	It occurred, and affected me A LOT	
10. I was told off during childbirth or my questions and doubts were answered disrespectfully (with criticism, yelling, or abuse)	9.64 (10.90)	14.40 (13.25)	19.73 (13.73)	24.62 (15.39)	**<0**.**001**
	It occurred	It occurred, but it did NOT affect me AT ALL	It occurred, and affected me A LITTLE	It occurred, and affected me A LOT	
11. They verbally scared or intimidated me about a danger to me or my baby into accepting certain practices that I did not agree with and they did NOT explain to me why they carried them out or with what justification	9.53 (10.60)	16.11 (16.00)	17.93 (13.43)	26.11 (15.54)	**<0**.**001**
	It occurred	It occurred, but it did NOT affect me AT ALL	It occurred, and affected me A LITTLE	It occurred, and affected me A LOT	
12. They spoke to me like I was a child or mocked me	9.75 (10.92)	14.22 (12.77)	20.13 (14.37)	27.01 (15.21)	**<0**.**001**
	It occurred	It occurred, but it did NOT affect me AT ALL	It occurred, and affected me A LITTLE	It occurred, and affected me A LOT	
13. I was criticized during childbirth for expressing my emotions (crying, yelling in pain, etc.)	10.01 (11.18)	15.79 (13.60)	18.42 (14.22)	25.69 (15.59)	**<0**.**001**
	It occurred	It occurred, but it did NOT affect me AT ALL	It occurred, and affected me A LITTLE	It occurred, and affected me A LOT	
14. During the birth experience, I was made to feel vulnerable, guilty, insecure, or that I had not lived up to what was expected of me (that I had not collaborated)	9.47 (10.57)	16.44 (13.03)	19.10 (14.15)	28.15 (15.24)	**<0**.**001**
Items on inadequate or innecessary procedures (Items 15–20)	They allowed me	They did not allow me, but it did not affect me AT ALL	They did not allow me, and it affected me A LITTLE	They did not allow me, and it affected me A LOT	
15. They allowed me to adopt the position that I requested during dilation and delivery when not contraindicated	9.79 (10.97)	13.89 (11.91)	16.46 (13.26)	27.15 (16.45)	**<0**.**001**
	Yes, they used it	They did not use it, but it did not affect me AT ALL	They did not use it, and it affected me A LITTLE	They did not use it, and it affected me A LOT	
16. They used anesthesia, whether requested or not, for example, to suture a tear or episiotomy or manually remove the placenta	10.88 (11.88)	12.04 (11.48)	20.59 (15.70)	25.57 (16.42)	**<0**.**001**
	Yes, they used measures	They did not use it, but it did not affect me AT ALL	They did not use it, and it affected me A LITTLE	They did not use it, and it affected me A LOT	
17. The vaginal examinations were performed on me without taking measures to reduce the discomfort that this entails (use of lubricant, performing the technique progressively, trying to relax)	9.42 (10.81)	14.85 (13.04)	15.64 (12.06)	25.78 (16.03)	**<0**.**001**
	Yes, with my consent	It occurred without my consent, but it did not affect me AT ALL	It occurred without my consent, and it affected me A LITTLE	It occurred without my consent, and it affected me A LOT	
18. They carried out some of these practices without my consent (enema, shaving, vaginal examinations, episiotomy, abdominal pressure)	9.80 (11.15)	13.48 (12.15)	15.97 (13.58)	23.85 (15.21)	**<0**.**001**
	It occurred	It occurred, but it did NOT affect me AT ALL	It occurred, and affected me A LITTLE	It occurred, and affected me A LOT	
19. I experienced some type of physical violence during labor. For example, I was slapped on the face or slapped on the thighs during childbirth to scold me or reprimand me for my behavior	11.16 (12.10)	13.47 (12.30)	25.78 (14.83)	33.90 (17.31)	**<0**.**001**
	They allowed it offered it	They did not allow it offer it, but it did not affect me AT ALL	They did not allow me/, and it affected me A LITTLE	They did not allow it offer it, but it did not affect me A LOT	
20.a I was allowed to do skin-to-skin immediately after giving birth without reasons and/or without giving explanations that would contraindicate it. (Only women with a live birth)	9.52 (10.71)	11.02 (10.62)	17.28 (13.79)	24.90 (15.17)	**<0**.**001**
20.b They offered me the possibility of seeing my baby or preparing a memory box. (Only women with fetal loss)					

*P* < 0.05 statistically significant difference (in bold).

### Risk of post-traumatic stress disorder (PTSD)

**Figure 2:**
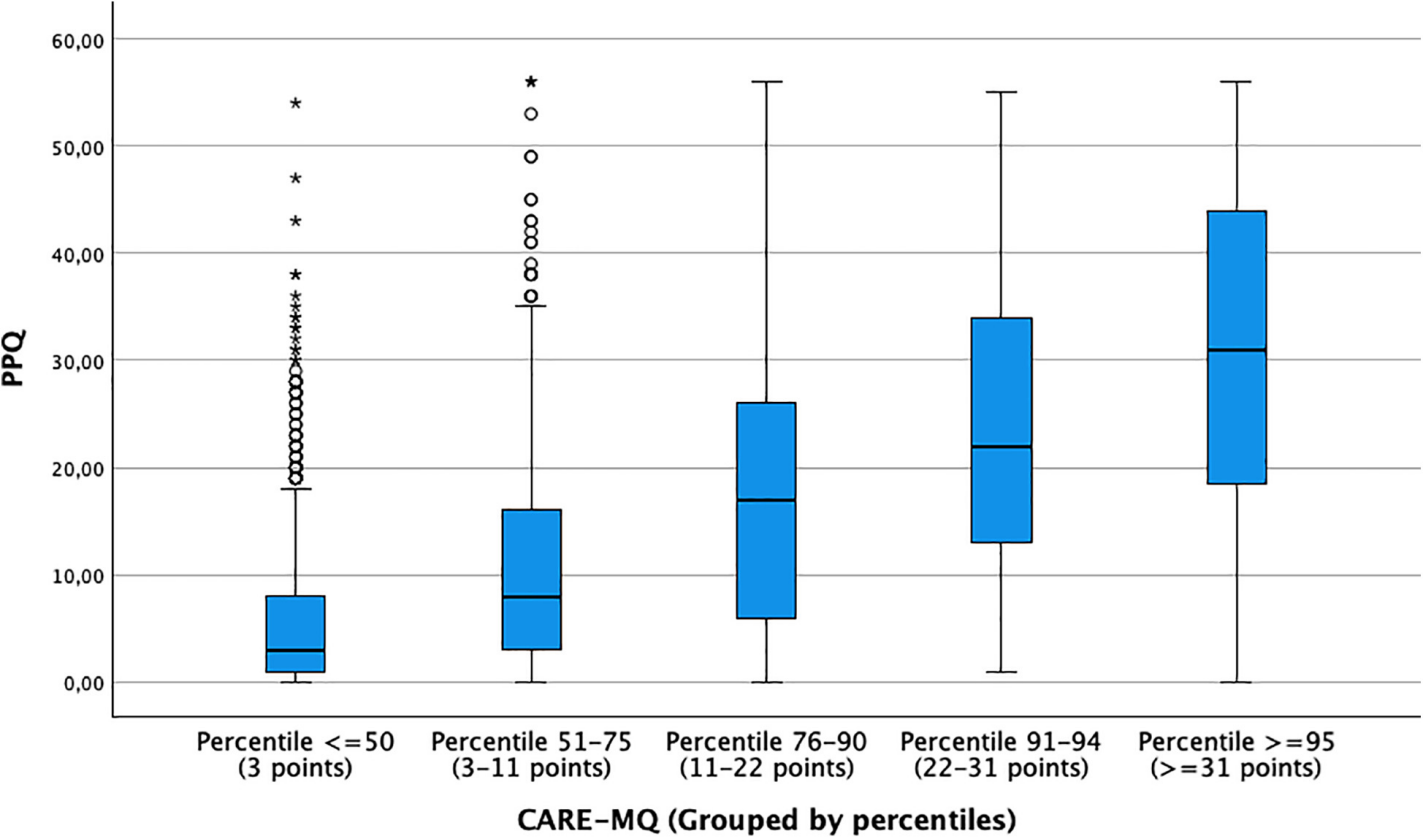
Relationship between CARE-MQ scores with PPQ scores grouped by percentiles to detect risk of post-traumatic stress disorder.

Finally, the relationship between the risk of PTSD and various sociodemographic and clinical factors of postpartum women was analyzed. As can be seen both in Figure 2 and in the bivariate and multivariate analysis (Table 3), there is a linear relationship between the highest CARE-MQ scores grouped by percentiles and the risk of PTSD. Thus, women with CARE-MQ scores over the 51th percentile had a greater probability of PTSD risk in a linear fashion. Women with a 51–75th percentile (medium level) had an aOR of 4.03 (95%CI: 2.25–7.21), for the 75th–90th percentile an aOR of 9.62 (95%CI: 5.36–17.28), for the 91–95th percentile an aOR of 19.15 (95%CI: 9.97–36.76) and for a percentile ≥95 an aOR of 34.72 (95%CI: 18.01–66.95) compared to those who had scores below the 50th percentile on the CARE-MQ.

**Table 3:** Factors associated with the risk of PSTD bivariate and multivariate analysis.

Variable	PTSD (PPQ > 90 percentile)		Bivariate analysis		Multivariate analysis	
	No	Yes	OR 95% CI	*P*-value	aOR 95% CI	*P*-value
	*n* (%)	*n* (%)				
	*N* = 2,619	*N* = 293				
CARE-MQ				**<0** **.** **001**		**<0**.**001**
Percentile <50 (3 points) little	1,322 (98.7)	17 (1.3)	1		1	
Percentile 51–75 (3–11 points) medium	754 (93.4)	53 (6.6)	**5.46** (**3.14–9.50)**	**<0**.**001**	**4.03** (**2.25–7.21)**	**<0**.**001**
Percentile 76–90 (11–22 points) high	370 (81.7)	83 (18.3)	**17.44** (**10.22–29.76)**	**<0**.**001**	**9.62** (**5.36–17.28)**	**<0**.**001**
Percentile 91–94 (22–31 points) very high	103 (64.0)	58 (36.0)	**43.79** (**24.60–77.94)**	**<0**.**001**	**19.15** (**9.97–36.76)**	**<0**.**001**
Percentile >95 (≧31 points) extreme	70 (46.1)	82 (53.9)	**91.09** (**51.26–161.88)**	**<0**.**001**	**34.72** (**18.01–66.95)**	**<0**.**001**
Maternal age mean (SD)	33.8 (3.98)	33.0 (4.46)	**0.95** (**0.93–0.99)**	**<0**.**001**	**0.94** (**0.91–0.98)**	**<0**.**001**
Family monthly income in euros				**<0**.**001**		
Less than 1,000 euros	33 (75.0)	11 (25.0)	1			
Between 1,000 and 1,900 euros/month	439 (85.7)	73 (14.3)	0.49 (0.24–1.03)	0.060		
Between 2,000 and 2,900 euros/month	926 (89.2)	112 (10.8)	**0.36** (**0.17–0.73)**	0.005		
Between 3,000 and 2,900 euros/month	743 (92.2)	63 (7.8)	**0.25** (**0.12–0.52)**	**<0**.**001**		
More than 4,000 euros	478 (93.4)	34 (6.6)	**0.21** (**0.09–0.45)**	**<0**.**001**		
Perception of amount of partner support				**<0**.**001**		**<0**.**001**
None	35 (71.4)	14 (28.6)	**1**		1	
Little	53 (74.6)	18 (25.4)	0.84 (0.37–1.92)	0.695	1.06 (0.40–2.81)	0.893
Some	153 (84.5)	28 (15.5)	**0.45** (**0.21–0.95)**	0.038	0.63 (0.26–1.54)	0.314
Sufficient	620 (87.9)	85 (12.1)	**0.34** (**0.17–0.66)**	**0**.**001**	0.58 (0.26–1.31)	0.196
A lot	1,758 (92.2)	148 (7.8)	**0.21** (**0.11–0.40)**	**<0**.**001**	**0.36** (**0.16–0.80)**	0.013
Planned pregnancy				**0**.**042**		
No	175 (85.8)	29 (14.2)	1			
Yes	2,444 (90.3)	264 (9.7)	**0.65** (**0.43–0.98)**			
Live fetus				**0**.**001**		
No	9 (60.0)	6 (40.0)	1			
Yes	2,610 (90.1)	287 (9.9)	**0.16** (**0.05–0.46)**			
Gestational age						**<0**.**001**
Term	2,489 (90.1)	272 (9.9)	1		1	
Moderate premature	118 (88.7)	15 (11.3)	1.16 (0.67–2.02)	0.591	0.69 (0.34–1.37)	0.294
Very premature	6 (100.0)	0 (0.0)	0	0.999	0	0.999
Extreme premature	6 (50.0)	6 (50.0)	**9.15** (**2.93–28.56)**	**<0**.**001**	**22.51** (**5.07–99.83)**	**<0**.**001**
Type of gestation				0.147		
Single	2,576 (90.0)	285 (10.0)	1			
Multiple	41 (83.7)	8 (16.3)	1.76 (0.81–3.79)			
Previous cesarean				**<0**.**001**		
No	2,013 (92.9)	155 (7.1)	1			
One	559 (80.8)	133 (19.2)	**3.09** (**2.40–3.96)**	**<0**.**001**		
Two or more	47 (90.4)	5 (9.6)	1.38 (0.54–3.52)	0.499		
Number of pregnancies				**0**.**004**		
One	1,629 (88.5)	211 (11.5)	1			
Two	724 (92.7)	57 (7.3)	**0.60** (**0.44–0.82)**	**0**.**001**		
Three or more	266 (91.4)	25 (8.6)	0.72 (0.47–1.12)	0.148		
Number of vaginal births				**<0**.**001**		
None	495 (79.6)	127 (20.4)	1			
One	1,668 (92.3)	140 (7.7)	**0.32** (**0.25–0.42)**	**<0**.**001**		
Two or more	456 (94.6)	26 (5.4)	**0.22** (**0.14–0.34)**	**<0**.**001**		
Number of miscarriages				0.732		
None	1,897 (90.1)	208 (9.9)	1			
One	531 (89.1)	65 (10.9)	1.11 (0.83–1.49)	0.464		
Two or more	191 (90.5)	20 (9.5)	0.95 (0.59–1.54)	0.852		
Hypertension				0.092		
No	2,393 (90.2)	259 (9.8)	1			
Yes	226 (86.9)	34 (13.1)	1.39 (0.94–2.03)			
Diabetes				0.406		
No	2,414 (90.1)	266 (9.9)	1			
Yes	205 (88.4)	27 (11.6)	1.19 (0.78–1.82)			
Spontaneous preterm birth				0.063		**0**.**020**
No	2,475 (90.2)	269 (9.8)	1		1	
Yes	144 (85.7)	24 (14.3)	1.53 (0.97–2.40)		**1.98** (**1.11–3.54)**	
Fertility treatment				0.142		
No	2,261 (89.6)	262 (10.4)	1			
Yes	358 (92.0)	31 (8.0)	0.74 (0.50–1.10)			
Parity				**0**.**002**		
Primiparous	2,018 (89.0)	249 (11.0)	1			
Multiparous	601 (93.2)	44 (6.8)	**0.59** (**0.42–0.82)**			
Antenatal classes				0.565		
No	418 (91.3)	40 (8.7)	1			
Yes, but less than 5 classes	505 (90.0)	56 (10.0)	1.15 (0.75–1.77)	0.498		
Yes at least 5 classes	1,696 (89.6)	197 (10.4)	1.21 (0.85–1.73)	0.287		
Induction of labor				**<0**.**001**		
No	1,449 (92.1)	125 (7.9)	1			
Yes	1,170 (87.4)	168 (12.6)	**1.66** (**1.30–2.12)**			
Birth plan				**<0**.**001**		**<0**.**001**
No	1,108 (91.7)	100 (8.3)	**1**		1	
Yes, but it wasn't respected	321 (67.6)	154 (32.4)	**5.31** (**4.01–7.03)**	**<0**.**001**	**1.66** (**1.18–2.34)**	**0**.**003**
Yes, and it was mostly respected	1,190 (96.8)	39 (3.2)	**0.36** (**0.24–0.53)**	**<0**.**001**	0.75 (0.49–1.14)	0.185
Problems during the last birth				**<0**.**001**		**<0**.**005**
No	2,074 (93.5)	145 (6.5)	1		1	
Yes	545 (78.6)	148 (21.4)	**3.88** (**3.03–4.97)**		**1.56** (**1.15–2.12)**	
Use of oxytocin				**0**.**013**		
No	1,092 (91.6)	100 (8.4)	1			
Yes	1,527 (88.8)	193 (11.2)	**1.38** (**1.07–1.77)**			
Use of regional anesthesia				**0**.**001**		
No	490 (93.9)	32 (6.1)	1			
Yes	2,129 (89.1)	261 (10.9)	**1.87** (**1.28–2.74)**			
Use of nitrous oxide				**<0**.**001**		
No	2,558 (90.6)	265 (9.4)	1			
Yes	61 (68.5)	28 (31.5)	**4.43** (**2.78–7.05)**			
General anesthesia				0.701		
No	2,498 (90.0)	278 (10.0)	1			
Yes	121 (89.0)	15 (11.0)	1.11 (0.64–1.93)			
Type of birth				**<0**.**001**		**<0**.**001**
Normal	1,581 (95.5)	74 (4.5)	1		1	
Assisted/Instrumental	514 (86.0)	84 (14.0)	**3.49** (**2.51–4.84)**	**<0**.**001**	**2.03** (**1.31–3.14)**	**0**.**001**
Planned cesarean section	113 (89.0)	14 (11.0)	**2.64** (**1.44–4.83)**	**0**.**002**	1.61 (0.79–3.26)	0.183
Emergency cesarean section	411 (77.3)	121 (22.7)	**6.29** (**4.61–8.56)**	**<0**.**001**	**2.30** (**1.56–3.38)**	**<0**.**001**
Episiotomy				0.710		
No	2,045 (90.0)	226 (10.0)	1			
Yes	574 (89.5)	67 (10.5)	1.05 (0.79–1.40)			
Severe tear				**<0**.**001**		
No	2,509 (90.4)	266 (9.6)	1			
Yes	110 (80.3)	27 (19.7)	**2.31** (**1.49–3.59)**			
Skin-to-skin				**<0**.**001**		
No	390 (73.0)	144 (27.0)	1			
Yes, but less than 50 min	292 (87.7)	41 (12.3)	**0.38** (**0.26–0.55)**	**<0**.**001**		
Yes, between 50 and 120 min	313 (93.2)	23 (6.8)	**0.19** (**0.12–0.31)**	**<0**.**001**		
Yes, at least 120 min	1,624 (95.0)	85 (5.0)	**0.14** (**0.10–0.18)**	**<0**.**001**		
Breastfeeding within first hour				**<0**.**001**		
No	637 (80.4)	155 (19.6)	1			
Yes	1,982 (93.5)	138 (6.5)	**0.28** (**0.22–0.36)**			
Newborn admission				**<0**.**001**		**0**.**003**
No	2,300 (91.1)	224 (8.9)	1		**1**	
Yes	319 (82.2)	69 (17.8)	**2.22** (**1.66–2.98)**		**1.75** (**1.21–2.55)**	
Maternal ICU admission				**0**.**004**		
No	2,582 (90.2)	282 (9.8)	1			
Yes	37 (77.1)	11 (22.9)	**2.72** (**1.37–5.39)**			
Hospital readmission				**0**.**005**		
No	2,559 (90.2)	278 (9.8)	1			
Yes	60 (80.0)	15 (20.0)	**2.30** (**1.29–4.10)**			
Postpartum surgical intervention				**<0**.**001**		0.070
No	2,494 (90.7)	257 (9.3)	1		1	
Yes	125 (77.6)	36 (22.4)	**2.79** (**1.88–4.13)**		**1.57 (0.96–2.57)**	

*P* < 0.05 statistically significant difference (in bold).

In this same analysis, the following factors were observed that increased the probability of presenting a risk of PTSD: Having an extremely premature birth (aOR:22.51; 95%CI: 5.07–99.83), the admission of the newborn (aOR: 1.75; 95%CI: 1.21–2.55) Having a birth plan not respected (aOR: 1.66; 95%CI: 1.18–2.34), had some type of problem during the last birth (aOR: 1.56; 95%CI: 1.15–2.12). A relationship was also observed with the type of birth; having an assisted/instrumental birth (aOR: 2.03; 95%CI: 1.31–3.14) and emergency cesarean section (aOR: 2.30; 95%CI: 1.56–3.38) increase the likelihood of PTSD relative to having a normal birth. On the other hand, at the oldest maternal age (aOR: 0.94; 95%CI: 0.91–0.98), the perception of a lot of support from their partner (aOR: 0.36; 95%CI: 0.16–0.80) was associated with a lower probability of PTSD risk.

## Discussion

The higher the scores on the CARE-MQ scale, the greater the risk of developing PTSD, as evaluated by the PPQ questionnaire. Of the four components of the CARE-MQ scale, inadequate treatment by professionals was the element that had the most correlation with PTDS. Among the risk factors identified, the following stand out: having an extremely premature birth, a birth plan that was not respected, having had some complication during the last birth, and the type of birth.

The detected prevalence of PTSD risk (>90th percentile) in our study was 10.1%. This is within the range found by Silva et al. (10) in a systematic review of factors associated with OV that are involved in the development of postpartum depression and PTSD. This systemic review included 21 studies, and a prevalence of PTSD between 0.3% and 24.5% was detected. Other studies obtained figures of between 4.7% and 11% risk for the general population, increasing to 15% in risk groups (16–18). This wide range of prevalence has been explained by differences in sampling, established cut-off points, or the timing of measurement in the different studies (20).

As previously described in the literature, the perception of OV constitutes a significant risk factor for the development of postpartum PTSD (25, 26). Our research findings show a linear relationship between perceptions of mistreatment and the risk of PTSD. In such a way that as scores on the CARE-MQ scale increase, scores on the PPQ scale also increase, suggesting that as the perception of disrespect and/or abuse during childbirth increases, the risk of developing postpartum PTSD also increases. Thus, women who were in the 51st–75th percentile of the CARE-MQ scale were 4 times more likely to develop postpartum PTSD, with the risk increasing 34 times if they were in the ≥95th percentile. Another relevant aspect of our study was knowing which dimension of the CARE-MQ scale correlated to the greatest extent with the risk of PTSD. Thus, we observed that inadequate treatment by professionals was the dimension that obtained the most correlation, followed by emotional abuse, family separation, and physical abuse. The “inappropriate treatment” dimension covers issues related to communication problems, privacy violation, and inappropriate or unnecessary techniques (24). In this sense, Leavy et al. (27) in their study on the relationship between OV and postpartum mental health, found that one of the aspects reported by women that influenced the risk of developing subsequent PTSD was inappropriate attitudes or behavior on the part of professionals during childbirth, increasing the degree of maternal dissatisfaction from 2.4% in the maternity ward to 6.5% 2 months postpartum. Van der Pijl et al. (28), in their study carried out in the Netherlands on the experience of childbirth in a sample of 12,239 women, showed that most of the time, they attributed a traumatic experience to the lack of options (39.8%) and lack of communication (29.9%). All of this confirms that actions and interactions with the health team constitute a key element in women's birth experience and this is a factor that health professionals can modify.

Regarding the identified risk factors, having an extremely premature birth and the admission of the newborn appear as risk factors for the development of PTSD. This may perhaps be because prematurity is a condition associated with greater complications on neonatal health, an aspect that can generate considerable emotional stress and anxiety in parents (29). This situation poses a risk to maternal health, as concluded in a recent systematic review on the prevalence of PTSD after admission to neonatal intensive care, where the presence of PTSD symptoms was observed in up to 40% of parents during the first month after birth (30).

Martínez et al. (14) concluded that having a birth plan that was not respected was a risk factor for the development of PTSD. Our results also reveal a significant relationship in accordance with what these researchers found, with the risk of suffering from PTSD being greater when not respecting women's preferences and needs in relation to their birth process. Along these lines, it has been shown that a birth plan increases the feeling of maternal control, reduces fear, and improves the birth experience (31), so its adherence becomes a protective factor and reduces the probability of PTSD symptoms (10, 26).

Another variable that emerges as a risk factor is having had a complication during the last birth. Also identified in previous research is an increased risk of PTSD with the existence of severe tears (14, 32–34), manual extraction of placenta (32, 35), non-reassuring patterns of fetal heart rate (14) and lack of skin-to-skin contact (36). In line with this, a qualitative study on women's perception of their birth experiences also identified this problem as factors that favor postpartum maternal trauma (37), largely associated with the pain experienced, fear, and need for information about the unforeseen events that may arise during the birth process.

Finally, numerous investigations agree that the type of birth impacts the subjective perception of the birth process (38). However, while cesarean sections may be necessary, it is the experience of an emergency cesarean section that has been more strongly linked to negative emotional outcomes and a higher risk of developing PTSD (14, 39). Similarly, assisted instrumental births are also associated with a significant risk factor for PTSD (10, 33, 34, 40). Our results align with these findings, as they indicate that the experience of an emergency cesarean section or an assisted instrumental birth constitutes a significant risk factor for the development of PTSD, compared to women who experience births without interventions. These results further support the recommendation of limiting non-emergency medical interventions to cases where they are strictly necessary.

This study is particularly significant for the following reasons. First, it stands out for using two validated questionnaires for data collection, which guarantees the reliability of the measurements and facilitates the interpretation of the results and their comparison with different populations or contexts. Second, it is a novel study that delves into the extent to which mistreatment and/or abuse during childbirth influences the development of PTSD risk.

As a limitation, there is a possible confounding bias inherent to the retrospective design of the study, as there may be difficulties in properly identifying and controlling confounding variables, although we performed a multivariate analysis to control it.

There is also a possibility of memory bias, particularly among women who experienced childbirth as traumatic. Since data were collected through retrospective self-reports, responses may have been influenced by participants' current emotional state or the lasting psychological impact of the event. While this bias does not invalidate the relevance of subjective perceptions of abuse, it should be considered when interpreting the findings.

Given the results obtained, it is essential to highlight the importance of continuous training for healthcare professionals—both in obstetric schools and clinical settings—in the field of perinatal mental health, with a specific focus on respectful, patient-centered care. This requires understanding the childbirth process from the woman's perspective, recognizing her emotions, expectations, and perceptions of safety and dignity. Educational programs should integrate training in empathetic communication, informed consent, and trauma-informed care, while institutional protocols in hospitals should support adherence to birth plans, promote women's autonomy, and encourage the humanization of care. The systematic use of validated tools such as the CARE-MQ can facilitate the early detection of potentially traumatic experiences and guide preventive interventions. Implementing these measures may significantly reduce the risk of postpartum post-traumatic stress disorder, while also improving maternal satisfaction, strengthening the patient-provider relationship, and enhancing both mental and physical postpartum outcomes. In this regard, our study provides empirical evidence to inform policy decisions and promote meaningful changes toward safer, more woman-centered maternity care.

## Data Availability

The original contributions presented in the study are included in the article/Supplementary Material, further inquiries can be directed to the corresponding author.
